# Comparison of Different Metrics for the Identification of Vascular Changes in Diabetic Retinopathy Using OCTA

**DOI:** 10.3389/fnins.2021.755730

**Published:** 2021-11-30

**Authors:** Luis Mendes, Inês P. Marques, José Cunha-Vaz

**Affiliations:** ^1^AIBILI, Association for Innovation and Biomedical Research on Light and Image, Coimbra, Portugal; ^2^Coimbra Institute for Clinical and Biomedical Research (iCBR), Faculty of Medicine, University of Coimbra, Coimbra, Portugal

**Keywords:** OCTA, diabetes, retinopathy, vascular, retina

## Abstract

Retinal vessel metrics identifying microvascular changes such as vessel closure (VC) have shown potential clinical value by identifying eyes with diabetic retinopathy (DR) at different severity levels and at increased risk for disease progression to more severe stages. We compare the performance of 11 different metrics, which include 2 metrics supplied by the manufacturer, based on OCTA for identification of VC in different Early Treatment for Diabetic Retinopathy Study (ETDRS) severity groups. OCTA en-face slabs from 84 healthy eyes (70 ± 4.8 years) and 78 eyes of diabetic individuals (67 ± 7.5 years) were processed using different methods that include abnormal intercapillary spaces (AIS), vessel density (VD), and nine metrics extracted from the en-face slab. The best separation between the eyes with DR and the control group was obtained in the superficial capillary plexus (SCP), with the full retina (FR) also performing well. In the SCP, the metrics that show better performance were the AIS and the VD with a value of area under curve (AUC) equal to 0.89 [95% CI 0.84–0.94] and 0.85 [95% CI 0.79–0.91], respectively, indicating that the VD metric supported by the manufacturer is satisfactory. The values of these metrics on the different ETDRS groups show a progressive increase in VC, which is correlated with disease severity.

## Introduction

Microvascular impairment, resulting in edema or ischemia, is a primary event in its pathogenesis, possibly in consequence of pathways activated by hyperglycemia or poor metabolic control (Cunha-Vaz et al., 2014). Indeed, we have demonstrated that its major manifestation is vessel closure (VC), representing small retinal vessels that are closed off (Marques et al., 2019, 2020; Santos et al., 2020). For many years, the reference method to perform the visualization of the retinal vasculature has been Fluorescein Angiography (FA). This 2D invasive technique requires the administration *via* an intravenous injection of the fluorescein sodium that can cause side effects like nausea and (rarely) anaphylaxis. The 2D nature of this technique does not allow the localization of the abnormalities on the 3D structure of the retina.

In the last few years, OCTA, a new technique that allows to visualize the 3D retinal vascular network, has emerged. This technique allows the quick capture of images of retinal and choroidal microvasculature that are registered with the structural information (OCT). Due to this non-invasive nature, it is possible to often perform this exam that allows the follow-up of the changes of the microvasculature. Despite the high costs associated and the need for a state-of-the-art computational infrastructure to store the volumetric information, it has been quickly adopted in clinical practice due to the benefits for the patients. Several metrics can be extracted with the objective to allow the clinicians to detect and follow-up the vascular alterations associated with diabetic retinopathy (DR) (Marques et al., 2020). These metrics include vessel density (VD; Zhang et al., 2016; Lei et al., 2017), perfusion density (PD; Lei et al., 2017), metrics related with the presence of intercapillary spaces (IS; Lauermann et al., 2019; Rocholz et al., 2019), and fractal dimension (FD; Zahid et al., 2016). However, currently, there is no established metric for the detection of VC.

In this study, we compare the performance of metrics that can be used in the detection and follow-up of VC on eyes with DR using OCTA. The range of tested metrics was extensive, comparing the methods supported by the manufacturer of the equipment with nine other metrics. All the metrics were computed based on the same en-face slab generated using the Carl Zeiss Meditec Density Exerciser (version:10.0.12787) and measured for the superficial capillary plexus (SCP), deep capillary plexus (SCP), and full retina (FR). The vessel and perfusion density were computed using the Carl Zeiss Meditec Density Exerciser, and the percentage of abnormal intercapillary spaces (AIS) was computed based on a method developed by the authors that explore two established image processing methods, the bottom-hat transform, and the closing morphological operator, to enhance the spaces of the binary version of the en-face image. An additional eight metrics were extracted from the en-face slab. These metrics include the FD and contrast of the en-face slab and histogram-based metrics after applying a Frangi filter to the en-face slab.

## Materials and Methods

### Data

The data used in this cross-sectional study were obtained from the data acquired in the study NCT03010397 designed to analyze eyes/patients with Type 2 Diabetes (T2D) and non-proliferative DR. The tenets of the Declaration of Helsinki were followed, approval was obtained from the AIBILI Ethics Committee for Health, and written informed consent to participate in the study was obtained from all individuals after all procedures were explained.

Exclusion criteria included any previous laser treatment or intravitreal injections, presence of other retinal diseases (e.g., age-related macular degeneration, glaucoma, or vitreomacular disease), high ametropia (spherical equivalent greater than –6 and + 2 diopters), or any other systemic disease that could affect the eye, with special attention for uncontrolled systemic hypertension (values outside normal range: systolic 70–210 mmHg and diastolic 50–120 mmHg) and history of ischemic heart disease.

The Early Treatment for Diabetic Retinopathy Study (ETDRS) disease severity scale is based on a modified version of the Airlie House classification system. The ETDRS classification is made using stereoscopic color fundus photography obtained from seven standard fields (30 degrees) and is the reference standard for grading diabetic retinopathy severity (ETDRS Report 10).

Seventy-eight eyes with DR and ETDRS scale between 10 and 53 were selected, with a maximum glycated hemoglobinA1c (HbA1c) value of 10%. An age-matched population including 84 eyes of individuals without diabetes or other retinal diseases was used as control group. Age, duration of diabetes, HbA1c, and blood pressure levels were collected for each participant at baseline visit.

OCT-Angiography data were collected by the Cirrus HD-OCT 5000 device (AngioPlex) using the Angiography 3 × 3 mm^2^ acquisition protocol, which is composed of a set of 245 clusters of four B-scans repetitions, where each B-scan consists of 245 A-scans, over a 3 × 3 × 2 mm^3^ volume in the central macula.

All OCTA examinations underwent a quality check to discard acquisitions having a signal strength lower than 7, motion artifacts, or evidence of defocus or blur in more than 25% of the area under analysis.

### En-Face Slabs

In this study, all the metrics were computed based on the en-face slabs generated by the Carl Zeiss Meditec Density Exerciser (version:10.0.12787).

**FIGURE 1:**
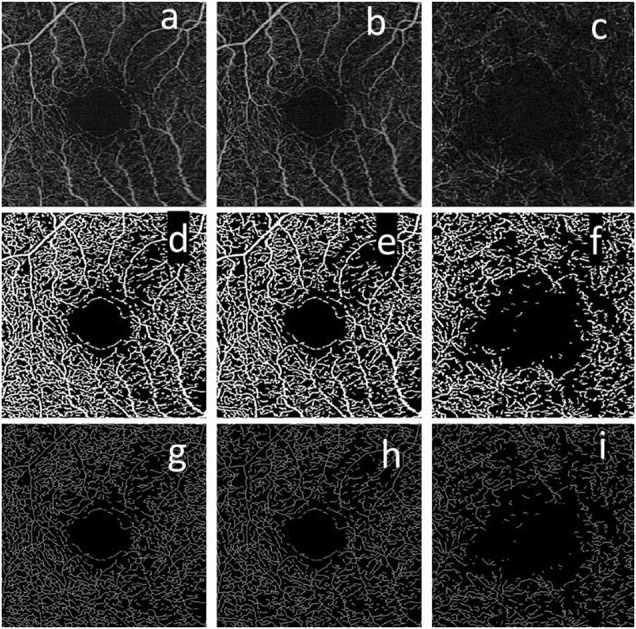
Example of the en-face slabs used to compute the vessel closure (VC) metrics. The top row corresponds to the en-face slab generated for the full retina (FR) **(a)**, superficial capillary plexus (SCP) **(b)**, and DCP **(c)**. The second row corresponds to the binary version of the en-face slabs for the FR **(d)**, SCP **(e)**, and DCP **(f)**. The last row corresponds to the skeletonized version of the en-face slab for the FR **(g)**, SCP **(h)**, and DCP **(i)**.

The metrics were calculated for the en-face slabs associated with the FR, SCP, and DCP en-face images. The inner and outer boundary for each slab were found using the following rules (Meditec, 2020):

• **FR en-face slab:** The inner boundary corresponds to the internal limiting membrane (ILM). The outer boundary is offset above the retinal pigment epithelium (RPE) by 70 microns to minimize the contribution of the hyper-reflective RPE. Figure 1a presents an example of this en-face slab.

• **SCP en-face slab:** The inner surface corresponds to the ILM. The outer surface approximates the inner plexiform layer (IPL), which is estimated by the following equation:

Z =IPLZ +ILM70%*(T −ILM)OPL

where Z_IPL_ is the boundary location of the estimated IPL, Z_ILM_ is the boundary location of the ILM, and T_ILM_-OPL is the thickness between ILM and the outer plexiform layer (OPL). Figure 1b presents an example of this en-face slab.

• **DCP en-face slab:** The inner surface is IPL as described above. The outer surface is OPL, which is approximated as:

Z=OPLZ-RPEfit110μ⁢m

where Z_OPL_ is the boundary location of the estimated OPL and Z_RPEfit_ is the boundary location of the RPE segmented in the same manner as CIRRUS HD-OCT structural images. Figure 1c presents an example of this en-face slab.

### Vessel Closure Metrics

#### Vessel and Perfusion Density

The Carl Zeiss Meditec Density Exerciser (version:10.0.12787) was used to calculate perfusion density (PD) and vessel density (VD). This software uses a thresholding algorithm to create a binary slab that assigns to each pixel a 1 (perfused) or 0 (background). Figures 1d–f present an example of these binary slabs for the FR, SCP, and DCP. Perfusion density is defined as the total area of perfused vasculature per unit area in a region of measurement, calculated by taking the mean of the binary slab within a desired region of interest, and represents changes in vessel caliber, namely, vasodilation, or vasoconstriction. Vessel density is the mean of the skeletonized slab within a desired region of interest, scaled by the distance between pixels (in this case, 245 pixels per 3 mm), and represents the number of individual vessels that are carrying red blood cells. Figures 1g–i present an example of the skeletonized binary slabs for the FR, SCP, and DCP.

#### Percentage of Abnormal Area of Intercapillary Spaces

**FIGURE 2:**
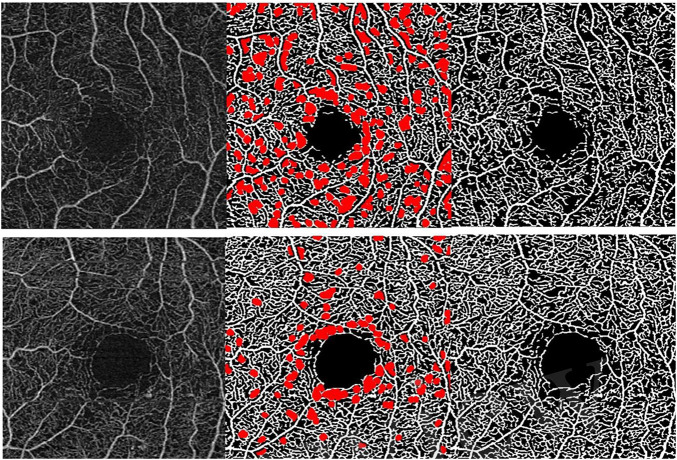
Two examples of regions detected with the abnormal intercapillary spaces (AIS) method. From the left to the right: SCP en-face slab acquired from eyes with Early Treatment for Diabetic Retinopathy Study (ETDRS) 35, the corresponding binary image highlighted with the detected AIS and the binary image.

A method that aims to detect the AIS was developed and implemented using the Wolfram Mathematica version 12.2. In this work, the percentage of the total area detected as abnormal was computed following the next steps:

(1) The bottom-hat transform is used to enhance the spaces of the binary slab of the en-face slabs generated by the Carl Zeiss Meditec Density Exerciser. The structuring element was a diamond matrix with the side equal to 4 pixels. The input image was resized to have 512 × 512 pixels.
(2) A closing morphological operation followed by an inversion of the binary values is applied to the enhanced image. The structuring element of the closing operation was a disk matrix with a radius equal to 5 pixels.
(3) The major vessels are removed from the resulting image of step 2 using a binary mask with the information of the localization of the major vessels. The binary mask was generated by detecting the 10% brightest pixels of the en-face slab image followed by applying a dilation operation with a structuring element box matrix with the side equal to 4 pixels.
(4) A matrix is generated in which each pixel of the image is replaced by an integer index representing the connected foreground image component in which the pixel lies.
(5) The component associated with the FAZ region is discarded and the total number of pixels is computed. Regions with area less than a threshold are discarded. In this work, the threshold was set to 100 pixels.
(6) The percentage of the area of intercapillary spaces is computed by dividing the total number of pixels associated with the intercapillary spaces by the total pixels of the slab. Figure 2 presents two examples of regions detected as AIS.

#### Entropy of the En-Face Image

This feature was computed using the Mathworks Matlab (R2020a) image processing toolbox.

#### Contrast (Haralick) of the En-Face Image

This feature was computed using the Wolfram Mathematica 12.2 image processing toolbox.

#### Fractal Dimension of the Binarized En-Face Image

This feature was computed based on an in-house implementation of the box counting method (Falconer, 2003) in Wolfram Mathematica 12.2.

#### Coarseness of the Tamura Filter of the En-Face Image

This feature was computed based on a Mathworks Matlab (R2020A) in-house implementation of the Coarseness, a measurement of the size of the primitive elements composing the texture, proposed by Tamura (Tamura et al., 1978).

#### Number of Pixels Assigned to Each Scale Generated During the Process of Applying a Frangi Filter to the En-Face Image

**FIGURE 3:**
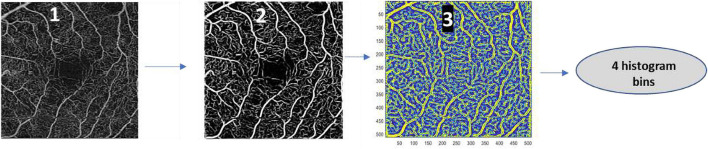
Steps done for the computation of the number of pixels assigned to each scale generated during the process of applying a Frangi Filter to the en-face image (histogram bins). Image 1 corresponds to the en-face slab image, image 2 corresponds to the enhanced vessel slab using the Frangi Filter, and image 3 corresponds to the matrix, resulting from the process of filtering the en-face slab with the Frangi Filter with four scales, with the scales on which the maximum intensity of every pixel is found.

The Frangi filter (Frangi et al., 1998), with four scales, was used to enhance the vessels of the en-face slab. In this study, we use as metric the number of pixels assigned to each scale of the Frangi filter on which the maximum intensity was found. The steps to compute these four metrics are presented in the Figure 3. A histogram of the metrics that contained the scale information for every pixel is used to compute these metrics. The Mathworks Matlab implementation of the Frangi filter used in this study is available at the Mathworks Matlab central (Kroon, 2021).

### Data Analysis

The area under curve (AUC) used to rank the metrics was computed using the Stata 16.1 (StataCorp LLC). The remaining statistics analysis and data cleaning were done using Wolfram Mathematica 12.2.

## Results

The rank (#) of the top five metrics having the AUC of the operating characteristic curve (ROC) as a criterion when the task was to separate eyes with DR (78 eyes) and the control group (84 eyes) is presented in Table 1. For each metric, the value of the AUC for the FR, SCP, and DCP is presented, as well as the associated 95% confidence interval. In Table 1, the metrics named FF_2 and FF_4 are associated with the scales of the Frangi filter on which the maximum intensity of every pixel is found. The FF_2 is associated to scale 2 and the FF_4 is associated to scale 4.

**TABLE 1:** Top five area under curve (AUC) values obtained when the task was to perform separation between eyes with diabetic retinopathy (DR) and control group.

	SCP		DCP		FR	
Feature	AUC	#	AUC	#	AUC	#
AIS	0.89 [0.84–0.94]	1	0.76 [0.69–0.83]	1	0.87 [0.81–0.93]	1
VD	0.85 [0.79–0.91]	2	0.65 [0.56–0.73]	7	0.80 [0.73–0.87]	2
FF_2	0.80 [0.73–0.87]	3	0.70 [0.62–0.78]	4	0.76 [0.69–0.84]	3
PD	0.76 [0.69–0.83]	4	0.65 [0.56–0.73]	6	0.73 [0.65–0.81]	4
FF_4	0.74 [0.66–0.81]	5	0.69 [0.61–0.77]	5	0.71[0.64–0.79]	5
CT	0.63 [0.55–0.72]	6	0.71 [0.63–0.79]	2	0.58 [0.50–0.67]	8
CTF	0.55 [0.46–0.64]	10	0.70 [0.62–0.79]	3	0.50 [0.41–0.59]	11

*The rank of the metric is also presented (#).*

The mean and the standard deviation of the top five metrics for the control group and the eyes belonging to the ETDRS 10–20 (24 patients) group, the 35 ETDRS group (31 patients), and the 43–53 ETDRS group (23 patients) measured at the FR, SCP, and DCP are presented in Table 2. Diabetic groups were identified with bold letters when the null hypothesis (the mean difference compared to the control group is 0) was rejected at the 5% level. Depending on the data distribution, the Mann–Whitney test (M) or the *t*-test (T) was used.

**TABLE 2:** Mean and standard deviation of the top five vessel closure (VC) metrics for the superficial capillary plexus (SCP) in the control group and eyes with DR by Early Treatment for Diabetic Retinopathy Study (ETDRS) grade.

SCP	Healthy	ETDRS 10–20	ETDRS 35	ETDRS 43–53
AIS	4.68 ± 1.4	**8.21 ± 4.7 (M)**	**9.27 ± 3.04(M)**	**10.76 ± 4.05(M)**
VD	21.06 ± 0.66	**19.71 ± 1.76 (M)**	**19.48 ± 1.21 (M)**	**18.85 ± 1.46 (M)**
FF_2	0.23 ± 0.01	**0.23 ± 0.01 (T)**	**0.23 ± 0.01 (T)**	**0.22 ± 0.01 (T)**
PD	0.38 ± 0.01	**0.36 ± 0.03 (T)**	**0.36 ± 0.02 (T)**	**0.35 ± 0.02 (T)**
FF_4	0.20 ± 0.01	**0.21 ± 0.01 (T)**	**0.21 ± 0.01 (T)**	**0.21 ± 0.01 (T)**

**DCP**	**Healthy**	**ETDRS 10–20**	**ETDRS 35**	**ETDRS 43–53**

AIS	11.28 ± 3.83	**15.6 ± 8.88 (M)**	**13.76 ± 3.41 (M)**	**17.05 ± 6.28 (M)**
CT	3.51 ± 0.48	**3.07 ± 0.54 (T)**	3.33 ± 0.56 (T)	**2.88 ± 0.43 (T)**
CTF	2.98 ± 0.06	**3.03 ± 0.09 (T)**	**3.03 ± 0.09 (T)**	**3.11 ± 0.1 (T)**
FF_2	0.25 ± 0.01	**0.24 ± 0.01 (T)**	**0.24 ± 0.01 (T)**	**0.24 ± 0.01 (T)**
FF_4	0.19 ± 0.01	**0.20 ± 0.01 (M)**	**0.20 ± 0.01 (T)**	**0.20 ± 0.01 (T)**

**FR**	**Healthy**	**ETDRS 10–20**	**ETDRS 35**	**ETDRS 43–53**

AIS	3.01 ± 1.09	**5.39 ± 3.59 (M)**	**5.88 ± 2.43 (M)**	**7.16 ± 3.07 (M)**
VD	22.44 ± 0.64	**21.40 ± 1.62 (M)**	**21.23 ± 1.23 (T)**	**20.52 ± 1.32 (T)**
FF_2	0.23 ± 0.01	**0.23 ± 0.01 (T)**	**0.23 ± 0.01 (T)**	**0.22 ± 0.01 (M)**
PD	0.40 ± 0.01	**0.38 ± 0.03 (T)**	**0.39 ± 0.02 (T)**	**0.38 ± 0.02 (M)**
FF_4	0.21 ± 0.01	**0.21 ± 0.01 (T)**	**0.22 ± 0.01 (T)**	**0.21 ± 0.01 (T)**

*The diabetic group was identified with bold letters when the null hypothesis, that the mean difference compared to the control group is 0, was rejected at the 5% level. Depending on the data distribution, the Mann–Whitney test (M) or the *t*-test (T) was used.*

As we can see in Figure 1, the vessels detected in the SCP and in the FR are similar, which suggests that the main contribution of vessels that the AngioPlex detects comes from the SCP. The top five metrics for the SCP (and FR) are the AIS (AUC equal to 0.89 [95% CI 0.84–0.94]), the VD (AUC equal to 0.85 [95% CI 0.79–0.91]), FF_2 (AUC equal to 0.80 [95% CI 0.73–0.87]), PD (AUC equal to 0.76 [95% CI 0.69–0.83]), and FF_4 (AUC equal to 0.74 [95% CI 0.66–0.81]). These results suggest that the percentage of the AIS and the number of vessels (VD) are the most discriminatory metrics.

## Discussion

In this study, we have examined the performance of 11 metrics extracted from en-face OCTA slabs to separate the VC measurements of 84 healthy eyes (70 ± 4.8 years) from measurements of 78 eyes of diabetic patients (67 ± 7.5 years). We also looked at the behavior of the metrics taking into account the progression of the disease. The ETDRS levels of the diabetic eyes included in our study range between 10 and 53: 24 patients with ETDRS 10–20, 31 patients with ETDRS 35, and 23 patients with ETDRS 43–53.

The rank of importance based on the AUC of these metrics was done for the FR, SCP, and DCP. The best separation between the eyes with DR, in the initial stages of DR, and the control group was obtained in the SCP, with the FR also obtaining a competitive performance (Table 1). This is particularly relevant as FR metrics include information from the entire retina and is independent of projection artifacts. Foveal VC has been considered to be a characteristic feature of the initial stages of diabetic retinal disease since the studies of Ashton and Cogan schools (Cogan and Kuwabara, 1963; Ashton, 1976). The decrease in VD and particularly with earlier detection in SCP indicates decrease in retinal blood flow in selected capillaries (Keith et al., 1967; Ludovico et al., 2003). These findings suggest that reduced capillary flow in the diabetic retina is related to a decrease in the number of capillaries that carry blood cells instead of changes in capillary diameter. The increase in VC, resulting from an increase in the number of capillaries that do not allow red blood cell flow was a result, at least in the beginning, of the development of preferential channels or arteriovenous shunts that are observed in histologic and trypsin-digested preparations of diabetic retinas (Cogan and Kuwabara, 1963; Keith et al., 1967).

Perfusion density gives information related to the total pixels associated with the vessels. The analysis of the rank of the VD and the PD may suggest that the additional information related to the caliber of vessels (included in the PD) does not introduce additional information that allows to improve the discrimination. In addition to the PD, the remaining two metrics that are in the top five ranks also encode information related to the caliber of the vessels. These metrics, FF_2 and FF_4, are associated with the scales of the Frangi filter on which the maximum intensity of every pixel is found. The values of the histogram bin associated with a lower scale 2 (FF_2) show better performance when compared with the higher scale 4 (FF_4), suggesting that the number of pixels associated with regions where the texture is noisier allows better discrimination when compared with the number of pixels associated with a rich texture.

For all top five metrics, we found statistically significant differences for all the three ETDRS groups when compared with the age match healthy group (Table 2). Although AIS allows a good separation between the eyes with DR and the control group, the value of standard variation associated with this metric is high. This behavior suggests that AIS may be sensitive to the image quality of the binary version of the en-face slab. Optimization of the two parameters of the method, the kernel sizes used in the bottom-hat transform and the kernel size of the morphological closing operation, may lead to obtaining more robust results (Figure 3). This optimization may be done using a cost function related to the improvement of the repeatability and reproducibility of the values of AIS. The optimization of the value of the threshold of the minimum area that is considered may also allow to improve the performance of the method.

It is considered highly relevant that the software analysis supplied by the manufacturer compares favorably with the other metrics tested. The values of all top five metrics on the ETDRS groups 10–20, 35, and 43–53 show a progressive increase in VC, which is correlated with the disease severity (Table 2). The results obtained with the different methods of analysis indicate that the microvascular changes in the initial stages of the DR are focal as indicated by the ranking of the AIS method, and involve predominantly closed capillaries (no flow capillaries) instead of changes in microvascular perfusion, i.e., vasoconstriction vs. vasodilatation, as shown by the better discrimination obtained with the VD vs. PD.

For the DCP, two different metrics appear in the top five rank: the contrast (CT) and the coarseness (CTF). As we can see in Figure 1, the vessels detected on the DCP are less defined when compared with the SCP. This behavior explains the lower values of AUCs and the improvement of the performance of the CT and the CTF. Eventually, an improvement in the segmentation used for the generation of the en-face slab may lead to an improvement in the overall quality of the en-face slab. The performance of the VD and PD on this capillary plexus decreased (are not in the top five ranks).

The main limitation of this study is that it is only valid for data acquired by the OCTA AngioPlex using the acquisition protocol Angiography 3 × 3. This limitation reflects a major problem regarding the comparison between OCTA metrics computed by the different equipment and the lack of standardization procedures that allow computing comparable values of the metrics. Although metrics like VD and PD are currently given by the equipment, on their development, the manufacturers adopted different methods/strategies that lead to the computation of no comparable values across the different equipment. Even in the same equipment, different acquisition protocols may lead to different values. These differences include the image formation algorithms, the acquisition protocol, the segmentation algorithm used for the generation of the en-face slab, and the methods used to compute the metrics. It must be realized also that the examiner experience and patient collaboration influence the final image quality and optimization of the signal/noise ratio. To achieve the objective of this research work, comparing the performance of the different methods that allow quantifying VC, the same slab was used as input to compute the values of the metrics. This approach ensures the fairness of the comparison.

## Conclusion

On OCTA AngioPlex, the measurement of VC shows better performance when analyzing the SCP using AIS or VD. These methods improve the discrimination of eyes with DR with different degrees of severity (ETDRS groups 10–20, 35, and 43–53), indicating that it is possible to identify non-proliferative DR severity using OCTA metrics with information obtained directly from the equipment.

## Data Availability Statement

The original contributions presented in the study are included in the article/supplementary material, further inquiries can be directed to the corresponding author.

## Ethics Statement

The studies involving human participants were reviewed and approved by the Association for Innovation and Biomedical Research on Light and Image Ethics Committee for Health. The patients/participants provided their written informed consent to participate in this study.

## Author Contributions

LM and IM collected and analyzed the data, and reviewed and edited the manuscript. JC-V analyzed the data and with LM discussed and wrote the manuscript. JC-V was the guarantor of this work and, as such, had full access to all the data in the study and takes responsibility for the integrity of the data and the accuracy of the data analysis. All authors contributed to the article and approved the submitted version.

## Conflict of Interest

JC-V reports grants from Carl Zeiss Meditec and is consultant for Alimera Sciences, Allergan, Bayer, Gene Signal, Novartis, Pfizer, Precision Ocular Ltd., Roche, Sanofi-Aventis, Vifor Pharma, and Carl Zeiss Meditec. The remaining authors declare that the research was conducted in the absence of any commercial or financial relationships that could be construed as a potential conflict of interest.

## Publisher’s Note

All claims expressed in this article are solely those of the authors and do not necessarily represent those of their affiliated organizations, or those of the publisher, the editors and the reviewers. Any product that may be evaluated in this article, or claim that may be made by its manufacturer, is not guaranteed or endorsed by the publisher.
